# The efficacy of calcium and vitamin D3 supplementation in improving pregnancy and neonatal outcomes

**DOI:** 10.3389/fped.2025.1605489

**Published:** 2025-06-12

**Authors:** Fei Wang, Mengqing Shen, Ting Wu, Yang Zhang, Yun Wang, Congyun Zhou

**Affiliations:** ^1^ Pediatrics and Child Health Department, Suzhou Ninth People’s Hospital, Suzhou, Jiangsu, China; ^2^ Department of Pharmacy, Ningbo No.6 Hospital, Ningbo, Zhejiang, China

**Keywords:** calcium, vitamin D, pregnancy, maternal health, neonatal outcomes

## Abstract

**Objective:**

This study aims to evaluate the effects of combined calcium and vitamin D3 supplementation on maternal, pregnancy, and neonatal outcomes.

**Methods:**

Clinical data from 122 pregnant women were retrospectively analyzed and categorized into three groups based on their actual intake: control (*n* = 48), Calcium 600-Vitamin D (600 mg calcium + 1,000 IU vitamin D daily, *n* = 41), and Calcium 1,200-Vitamin D (1,200 mg calcium + 1,000 IU vitamin D daily, *n* = 33). Baseline characteristics and outcomes including gestational diabetes, preeclampsia, gestational hypertension, birth weight, and Apgar scores were collected and analyzed.

**Results:**

Calcium and vitamin D₃ supplementation was associated with significantly lower rates of gestational diabetes (*P* = 0.035), preeclampsia (*P* = 0.032), and gestational hypertension (*P* = 0.047), as well as reduced leg cramp frequency (*P* < 0.001). Neonatal outcomes improved with higher birth weights (*P* < 0.001) and better 1-minute Apgar scores (*P* < 0.001). Multivariable logistic regression confirmed that high-dose supplementation (Calcium 1,200–Vitamin D) was independently associated with reduced risks of gestational diabetes (OR = 0.423, 95% CI: 0.123–0.869, *P* = 0.043) and gestational hypertension (OR = 0.342, 95% CI: 0.126–0.875, *P* = 0.012). Both supplementation groups significantly reduced leg cramp frequency: Calcium 600–Vitamin D (OR = 0.507, 95% CI: 0.355–0.723, *P* < 0.001) and Calcium 1,200–Vitamin D (OR = 0.512, 95% CI: 0.256–0.985, *P* = 0.012). No significant differences were found in other outcomes including preterm birth, neonatal infection, or NICU admission (*P* > 0.05).

**Conclusion:**

This study suggests that combined calcium and vitamin D₃ supplementation during pregnancy may help reduce the risk of pregnancy-related complications and improve neonatal outcomes, supporting its potential as an adjunct to prenatal care. Further large-scale and long-term studies are warranted to confirm these findings.

## Introduction

Calcium and vitamin D are essential nutrients that play critical roles in various physiological processes, including bone health, immune function, and cellular metabolism (1). During pregnancy, the demand for these nutrients increases significantly to support fetal development and maternal health (2, 3). Calcium is crucial for the development of the fetal skeleton, while vitamin D facilitates calcium absorption and maintains proper calcium and phosphate balance in the body (4). Deficiencies in either nutrient can lead to adverse health outcomes for both the mother and the fetus, making adequate intake during pregnancy particularly important (4).

Despite the well-established importance of calcium and vitamin D, deficiencies remain common among pregnant women worldwide (5, 6). Factors such as dietary inadequacies, limited sun exposure, and increased physiological demands contribute to the high prevalence of these deficiencies (6). Inadequate calcium intake during pregnancy has been associated with increased risks of hypertensive disorders, including preeclampsia (7), while vitamin D deficiency has been linked to gestational diabetes, preterm birth, and impaired fetal growth (8). These complications underscore the need for effective strategies to ensure sufficient intake of these nutrients during pregnancy.

Previous studies have highlighted the clinical significance of calcium and vitamin D supplementation. For instance, Kinshella et al. (9) have demonstrated that calcium supplementation during pregnancy reduced the risk of preeclampsia by 52% and significantly lowered the incidence of preterm birth. Another study by Yue and Ying (10) has found that vitamin D supplementation in pregnant women led to a high reduction in the risk of gestational diabetes and improved glucose metabolism. Furthermore, Wagner et al. (11) have reported that higher doses of vitamin D supplementation were associated with improved birth outcomes, including higher birth weights and lower rates of neonatal complications.

However, the optimal dosages and combined effects of these supplements remain areas of ongoing research. Additionally, few studies have comprehensively evaluated the combined supplementation of calcium and vitamin D at different dosages, particularly in populations with a high prevalence of deficiencies.

This study aims to evaluate the effects of combined calcium and vitamin D supplementation at two different dosages on maternal, pregnancy, and neonatal outcomes. By comparing outcomes among pregnant women receiving no supplementation, those receiving a moderate dose of calcium and vitamin D, and those receiving a higher dose, we aim to determine the optimal supplementation strategy to improve health outcomes for both mothers and their infants. The findings of this study could play a pivotal role in shaping future nutritional recommendations and ensuring that all pregnant women receive the nutrients they need for a healthy pregnancy and optimal fetal development.

## Methods

### Study design and participants

This study was conducted at Suzhou Ninth People's Hospital and retrospectively analyzed the clinical data of 122 pregnant women who underwent prenatal check-ups between May 2023 and April 2024. They were divided into three groups according to the actual dose of calcium and vitamin D3 supplementation: the control group (*n* = 48), the Calcium 600-Vitamin D group (*n* = 41), and the Calcium 1,200-Vitamin D group (*n* = 33). The study protocol was approved by the Ethics Committee of Suzhou Ninth People's Hospital (KY-2023004). All subjects signed the consent form before participation in the study.

Inclusion criteria: healthy pregnant women; aged 20–40 years; with a singleton pregnancy of at least 20 weeks gestation; no severe complications or chronic diseases; no calcium or vitamin D metabolic disorders; and the ability to comply with the treatment protocol. Exclusion criteria: multiple pregnancies; Women with pre-existing hypertension/diabetes or complications diagnosed before supplementation initiation were excluded to isolate supplement effects on incident outcomes; a history of severe pregnancy complications; use of diuretics or calcium channel blockers; and allergies to the study medications.

### Intervention

Participants in the control group did not receive any calcium or vitamin D supplements. Those in the Calcium 600-Vitamin D group were given 600 mg of calcium and 1,000 IU of vitamin D daily, while participants in the Calcium 1,200-Vitamin D group received 1,200 mg of calcium and 1,000 IU of vitamin D daily. The supplementation continued from enrollment until delivery. Compliance with the supplementation regimen was monitored through regular follow-ups. The three regimens reflected: (1) Standard care (control), (2) WHO-recommended pregnancy dose (600 mg/1,000 IU), and (3) High-dose for women with baseline insufficiency [serum 25(OH)D < 30 nmol/L or calcium < 2.1 mmol/L]. Allocation was based on baseline nutritional status.

### Data collection

Clinical baseline data were collected, including age, gestational age, parity (nulliparous or multiparous), smoking status (non-smoker or smoker), weight gain during pregnancy (categorized as inadequate, adequate, or excessive), and physical activity levels before and during pregnancy (classified as none, low, moderate, or high intensity).

Maternal outcomes were assessed by recording the incidence of gestational diabetes, preeclampsia, gestational hypertension, and weekly frequency of leg cramps. Blood pressure [Systolic Blood Pressure (SBP) and Diastolic Blood Pressure (DBP)], blood calcium levels, and vitamin D levels were measured at baseline and at delivery.

Pregnancy and neonatal outcomes were thoroughly documented, including miscarriage rates, live birth rates, mode of delivery (vaginal delivery or cesarean section), preterm birth, premature rupture of membranes, chorioamnionitis, polyhydramnios, gestational age, neonatal sex, birth weight, birth length, head circumference, 1- and 5-minute Apgar scores, neonatal asphyxia, neonatal infection, and Neonatal Intensive Care Unit (NICU) admissions. The Apgar scores, which evaluate the health of newborns at 1 and 5 min after birth, were assessed based on five criteria: heart rate, respiratory effort, muscle tone, reflex response, and color, with each criterion scoring between 0 and 2, and a total score ranging from 0 to 10 (12).

The occurrence of adverse effects such as fever, diarrhea, rash, joint pain, and dizziness/headache was monitored throughout the study period. These adverse effects were recorded based on self-reports from participants and verified through clinical assessments.

### Statistical analyses

Statistical analyses were conducted using SPSS 24.0 (SPSS Inc., Chicago, IL, USA). Continuous variables were expressed as mean ± SD and compared using ANOVA or the Kruskal–Wallis test, based on Shapiro–Wilk normality results. *Post-hoc* comparisons were performed using the Dunnett test. Categorical variables were analyzed using the Chi-square or Fisher's exact test. Multivariable logistic regression assessed associations between supplementation and binary outcomes (e.g., GDM, gestational hypertension, NICU admission), adjusting for maternal age, parity, gestational weight gain, smoking, and physical activity. Results were presented as odds ratios (ORs) with 95% confidence intervals, and *P* < 0.05 was considered significant.

## Results

### Baseline characteristics

The baseline characteristics of the participants are summarized in Table 1. There were no significant differences among the three groups regarding age, gestational age, parity, smoking status, weight gain during pregnancy, physical activity levels before and during pregnancy, Dietary Calcium, and Dietary Vitamin D (*P* > 0.05 for all comparisons).

**Table 1:** Baseline characteristics of participants.

Characteristics	Control group (*n* = 48)	Calcium 600-vitamin D group (*n* = 41)	Calcium 1,200-vitamin D group (*n* = 33)	*P*
Age (years)	28.94 ± 5.98	29.00 ± 5.39	29.36 ± 5.53	0.941
Gestational age (weeks)	38.60 ± 0.54	38.66 ± 0.57	38.42 ± 0.66	0.212
Parity				0.981
Nulliparous	38 (79.2)	33 (80.5)	26 (78.8)	
Multiparous	10 (20.8)	8 (19.5)	7 (21.2)	
Smoking status				0.913
Non-smokers	42 (87.5)	37 (90.2)	29 (87.9)	
Smokers	6 (12.5)	4 (9.8)	4 (12.1)	
Weight gain during pregnancy				0.748
Inadequate	3 (6.3)	2 (4.9)	1 (3.0)	
Adequate	42 (87.5)	38 (92.7)	29 (87.9)	
Excessive	3 (6.3)	1 (2.4)	3 (9.1)	
Physical activity pre-pregnancy				0.191
None	4 (8.3)	0 (0.0)	4 (12.1)	
Low	38 (79.2)	31 (75.6)	22 (66.7)	
Moderate	3 (6.3)	6 (14.6)	6 (18.2)	
High	3 (6.3)	4 (9.8)	1 (3.0)	
Physical activity during pregnancy				0.944
None	1 (2.1)	1 (2.4)	1 (3.0)	
Low	40 (83.3)	35 (85.4)	25 (75.8)	
Moderate	3 (6.3)	3 (7.3)	4 (12.1)	
High	4 (8.3)	2 (4.9)	3 (9.1)	
Dietary calcium (mg/day)	1,127 ± 300	1,098 ± 244	1,027 ± 280	0.275
Dietary vitamin D (IU/day)	448 ± 200	494 ± 147	453 ± 136	0.393

*P* < 0.05 represents statistically significance.

### Maternal outcomes

As shown in Table 2, the supplementation of calcium and vitamin D3 had significant effects on several maternal outcomes. The incidence of gestational diabetes was significantly lower in both the Calcium 600-Vitamin D group (7.3%) and the Calcium 1,200-Vitamin D group (6.1%) compared to the control group (22.9%) (*P* = 0.035). Similarly, preeclampsia rates were reduced to 2.4% and 0% in the Calcium 600-Vitamin D and Calcium 1,200-Vitamin D groups, respectively, vs. 12.5% in the control group (*P* = 0.032). Gestational hypertension was significantly lower in the supplemented groups, with rates of 9.8% and 0%, compared to 16.7% in the control group (*P* = 0.047). The frequency of leg cramps per week was also significantly reduced in the Calcium 600-Vitamin D and Calcium 1,200-Vitamin D groups (*P* < 0.001 for both comparisons). There were no significant differences in baseline SBP and DBP among the groups (*P* > 0.05), but at delivery, both SBP and DBP were significantly lower in the Calcium 600-Vitamin D and Calcium 1,200-Vitamin D groups (*P* < 0.05 for both).

**Table 2:** Effects of calcium-vitamin D3 supplementation on maternal outcomes.

Outcome	Control group (*n* = 48)	Calcium 600-vitamin D group (*n* = 41)	Calcium 1,200-vitamin D group (*n* = 33)	*P*
Gestational diabetes	11 (22.9)	3 (7.3)	2 (6.1)	0.035
Preeclampsia	6 (12.5)	1 (2.4)	0 (0.0)	0.032
Gestational hypertension	8 (16.7)	4 (9.8)	0 (0.0)	0.047
Leg cramps frequency (per week)	5.40 ± 2.35	3.32 ± 2.04	2.52 ± 1.35	<0.001
SBP (mmHg)	115.02 ± 11.11	116.98 ± 9.90	120.88 ± 11.63	0.058
Baseline	137.67 ± 12.02	132.49 ± 10.95^a^	127.18 ± 9.19^b^	<0.001
At delivery				
DBP (mmHg)	72.60 ± 8.27	72.17 ± 7.85	70.09 ± 8.03	0.338
Baseline	85.33 ± 8.77	79.2 ± 7.39^a^	78.06 ± 9.25^a^	0.001
At delivery				
Blood calcium (mmol/L)	1.76 ± 0.94	1.68 ± 0.75	2.09 ± 0.89	0.112
Baseline	1.54 ± 0.83	1.99 ± 0.84^a^	2.41 ± 0.99^b^	<0.001
At delivery				
Vitamin D (ng/ml)	9.95 ± 1.63	9.96 ± 1.44	10.47 ± 1.18	0.288
Baseline	10.50 ± 2.10	11.78 ± 1.50^a^	12.44 ± 1.76^a^	<0.001
At delivery	11 (22.9)	3 (7.3)	2 (6.1)	0.035

SBP, systolic blood pressure; DBP, diastolic blood pressure. *P* used Dunnett methods. *P* < 0.05 represents statistically significance.

^a^ *P* < 0.05 vs. control group.

^b^ *P* < 0.05 vs. calcium 600-vitamin D Group.

### Pregnancy and neonatal outcomes

The effects of calcium and vitamin D3 supplementation on pregnancy and neonatal outcomes are detailed in Table 3. The miscarriage rate was significantly lower in both the Calcium 600-Vitamin D (2.4%) and Calcium 1,200-Vitamin D (3.0%) groups compared to the control group (14.6%) (*P* = 0.049). The mode of delivery also differed significantly, with a higher rate of vaginal deliveries in the Calcium 600-Vitamin D (87.5%) and Calcium 1,200-Vitamin D (84.4%) groups compared to the control group (65.9%) (*P* = 0.039). The preterm birth rate was significantly lower in the supplemented groups (5.0% and 3.1%) compared to the control group (19.5%) (*P* = 0.029). The incidence of polyhydramnios was reduced to 2.5% and 3.1% in the Calcium 600-Vitamin D and Calcium 1,200-Vitamin D groups, significantly lower than the control group's 19.5% (*P* = 0.011). Other pregnancy complications, such as premature rupture of membranes and chorioamnionitis, did not show significant differences among the groups (*P* > 0.05). Regarding neonatal outcomes, birth weight was significantly higher in the Calcium 600-Vitamin D (3,413.53 ± 251.29 g) and Calcium 1,200-Vitamin D (3,561.78 ± 230.64 g) groups compared to the control group (3,179.54 ± 409.81 g) (*P* < 0.001). Additionally, the 1-minute Apgar score was significantly higher in the supplemented groups (*P* = 0.001), though the 5-minute Apgar score and the incidence of neonatal asphyxia did not differ significantly (*P* > 0.05).

**Table 3:** Effects of calcium-vitamin D3 supplementation on pregnancy and neonatal outcomes.

Outcome	Control Group (*n* = 48)	Calcium 600-Vitamin D Group (*n* = 41)	Calcium 1,200-Vitamin D Group (*n* = 33)	*P*
Miscarriage rate	7 (14.6)	1 (2.4)^a^	1 (3.0)^a^	0.049
Live birth rate	41 (100.0)	40 (100.0)	32 (100.0)	-
Mode of delivery				0.039
Vaginal delivery	27 (65.9)	35 (87.5)	27 (84.4)	
Cesarean section	14 (34.1)	5 (12.5)	5 (15.6)	
Preterm birth	8 (19.5)	2 (5.0)^a^	1 (3.1)^a^	0.029
Premature rupture of membranes	4 (9.8)	2 (5.0)	2 (6.3)	0.690
Chorioamnionitis	3 (7.3)	1 (2.5)	1 (3.1)	0.525
Polyhydramnios	8 (19.5)	1 (2.5)^a^	1 (3.1)^a^	0.011
Neonatal outcomes				0.977
Male	30 (73.2)	30 (75.0)	24 (75.0)	
Female	11 (26.8)	10 (25.0)	8 (25.0)	
Gestational age (weeks)	36.1 ± 3.44	38.33 ± 1.75^a^	37.97 ± 1.84^a^	<0.001
Birth weight (g)	3,179.54 ± 409.81	3,413.53 ± 251.29^a^	3,561.78 ± 230.64^b^	<0.001
Birth length (cm)	49.5 ± 2.01	50.09 ± 2.13	50.19 ± 2.28	0.252
Head circumference (cm)	35.64 ± 2.11	36.23 ± 2.04	35.32 ± 2.26	0.168
1-minute Apgar score	6.02 ± 2.24	7.15 ± 0.97	7.22 ± 0.91	<0.001
5-minute Apgar score	9.85 ± 1.13	9.70 ± 1.22	9.75 ± 1.05	0.828
Neonatal asphyxia	1 (2.4)	0 (0.0)	0 (0.0)	0.314
Neonatal infection	7 (17.1)	1 (2.5)^a^	1 (2.5)^a^	0.026
NICU admission	5 (12.2)	1 (2.5)^a^	0 (0.0)^a^	0.043

NICU, neonatal intensive care unit. *P* used Dunnett methods. *P* < 0.05 represents statistically significance.

^a^ *P* < 0.05 vs. control group.

^b^ *P* < 0.05 vs. calcium 600-vitamin D group.

### Multivariable logistic regression analyses

To assess the independent effect of supplementation, multivariable logistic regression was performed, adjusting for maternal age, parity, gestational weight gain, smoking status, and physical activity. After adjustment, Calcium 1,000-Vitamin D supplementation was significantly associated with reduced odds of gestational diabetes (OR = 0.423, 95% CI: 0.123–0.869, *P* = 0.043) and gestational hypertension (aOR = 0.342, 95% CI: 0.126–0.875, *P* = 0.012), compared to the control group. The Calcium 600–Vitamin D group showed similar trends, with a borderline association for gestational diabetes (OR = 0.136, 95% CI: 0.018–1.022, *P* = 0.053). Both supplementation groups were also significantly associated reduced weekly leg cramp frequency (Calcium 600: OR = 0.507, 95% CI: 0.355–0.723; Calcium 1,200: OR = 0.512, 95% CI: 0.256–0.985; *P* < 0.001). No significant associations were observed for preeclampsia, 1-minute Apgar score, neonatal infection, or NICU admission, although the effect estimates generally favored supplementation. Full results are presented in Table 4.

**Table 4:** Multivariable logistic regression analysis of maternal and neonatal outcomes associated with calcium and vitamin D supplementation.

Outcome	Calcium 600-vitamin D vs. control		Calcium 1,000-vitamin D vs. control	
	aOR (95% CI)	*P*	aOR (95% CI)	*P*
Gestational diabetes mellitus	0.136 (0.018–1.022)	0.053	0.423 (0.123–0.869)	0.043
Preeclampsia	0.024 (0.000–2.457)	0.115	0.785 (0.562–1.526)	0.089
Gestational hypertension	0.184 (0.021–1.599)	0.125	0.342 (0.126–0.875)	0.012
Leg cramps (frequency/week)	0.507 (0.355–0.723)	0.000	0.512 (0.256–0.985)	0.000
Gestational age	1.315 (0.864–2.003)	0.201	1.201 (0.596–2.132)	0.156
Neonatal birth weight (g)	1.003 (0.999–1.006)	0.132	1.245 (0.569–3.263)	0.263
1-minute Apgar score	1.512 (0.6989–2.312)	0.057	1.333 (0.562–1.956)	0.123
Neonatal infection	0.376 (0.032–4.433)	0.437	1.523 (0.562–3.263)	0.423
NICU admission	0.084 (0.005–1.356)	0.081	2.123 (0.452–5.699)	0.486

OR, adjusted odds ratio; CI, confidence interval. Logistic regression models adjusted for maternal age, parity, gestational weight gain, smoking status, and physical activity level. *P* < 0.05 represents statistically significance.

### Adverse effects

In addition to clinical efficacy, the tolerability and adverse effects of the interventions were assessed. The adverse effects of calcium and vitamin D3 supplementation are summarized in Table 5. There were no significant differences in the incidence of fever, diarrhea, rash, joint pain, and dizziness/headache among the control, Calcium 600-Vitamin D, and Calcium 1,200-Vitamin D groups (*P* > 0.05 for all comparisons). The occurrence of these adverse effects was low and comparable across all groups, indicating that calcium and vitamin D3 supplementation was well-tolerated by the participants. Notably, constipation—a frequently reported adverse effect of calcium supplementation—was comparably low across groups (Control: 8.3% vs. Calcium 600: 7.3% vs. Calcium 1,200: 6.1%, *P* = 0.928), indicating the regimens were well-tolerated.

**Table 5:** Adverse effects of calcium-vitamin D3 supplementation in pregnant women.

Adverse effect	Control group (*n* = 48)	Calcium 600-vitamin D group (*n* = 41)	Calcium 1,200-vitamin D group (*n* = 33)	*P*
Fever	2 (4.2)	1 (2.4)	1 (3.0)	0.897
Diarrhea	2 (4.2)	1 (2.4)	1 (3.0)	0.897
Rash	1 (2.1)	1 (2.4)	1 (3.0)	0.964
Joint pain	3 (6.3)	1 (2.4)	0 (0.0)	0.280
Dizziness/Headache	4 (8.3)	1 (2.4)	1 (3.0)	0.370
Constipation	4 (8.3)	3 (7.3)	2 (6.1)	0.928

*P* < 0.05 represents statistically significance.

## Discussion

This study demonstrates that combined calcium and vitamin D3 supplementation significantly improves maternal, pregnancy, and neonatal outcomes among pregnant women. The findings provide compelling evidence for the benefits of such supplementation, particularly in reducing the incidence of gestational diabetes, preeclampsia, gestational hypertension, and preterm birth. The results also indicate improved neonatal outcomes, such as higher birth weights and better Apgar scores, which are critical indicators of newborn health.

The beneficial effects of calcium and vitamin D supplementation may be attributed to their essential roles in various physiological processes (13, 14). Calcium is critical for maintaining vascular smooth muscle tone and endothelial function, which may contribute to the reduced risk of gestational hypertension and preeclampsia (15, 16). Vitamin D facilitates intestinal calcium absorption and modulates immune function, potentially offering protective effects against gestational diabetes and preeclampsia (17). Moreover, the active form of vitamin D, 1,25-dihydroxyvitamin D₃, regulates placental calcium and phosphate transport, which is essential for fetal skeletal mineralization. Dysregulation of vitamin D metabolism—such as downregulation of CYP2R1 and VDR—has been linked to gestational diabetes and preeclampsia and may impair calcium signaling and increase oxidative stress, thereby reducing neonatal bone mineral content (18). Insufficient calcium and vitamin D intake may also disrupt placental morphogenesis, potentially elevating the risk of preterm birth. Therefore, combined supplementation may improve maternal and neonatal outcomes through multiple mechanisms, supporting the clinical benefits observed in this study (15).

Our study adds to the growing body of evidence linking calcium and vitamin D deficiencies to adverse pregnancy outcomes. The significant reduction in the incidence of gestational diabetes and preeclampsia with supplementation underscores the importance of addressing these deficiencies (19). Gestational diabetes and preeclampsia are serious conditions that can lead to long-term health issues for both mother and child, including an increased risk of type 2 diabetes and cardiovascular diseases (20). By mitigating these risks, calcium and vitamin D supplementation has the potential to improve long-term health outcomes significantly (21).

Our findings are consistent with several previous studies that have reported the benefits of calcium and vitamin D supplementation during pregnancy. For instance, a study by Wang et al. (22) has found that vitamin D supplementation significantly reduced the risk of gestational diabetes and improved glucose metabolism in pregnant women. Similarly, another study by Gomes et al. (23) has demonstrated that calcium supplementation reduced the risk of preeclampsia and hypertensive disorders in pregnant women. However, our study goes further by evaluating the combined effects of these supplements at different dosages, providing more nuanced insights into the optimal levels required for maximal benefit.

In contrast, some studies have reported no significant benefits of calcium and vitamin D supplementation, which could be due to differences in study design, population characteristics, and supplementation protocols (24, 25). For example, the lack of effect in some studies might be attributed to lower baseline deficiencies in the study populations or variations in the timing and duration of supplementation (26). Our study's rigorous design and comprehensive data collection contribute to a more robust understanding of these nutrients' impact during pregnancy.

Furthermore, our multivariable logistic regression analysis confirmed that the observed benefits of supplementation were not merely attributable to baseline differences among participants. After controlling for potential confounders—maternal age, parity, gestational weight gain, smoking status, and physical activity—high-dose calcium and vitamin D supplementation remained significantly associated with reduced risks of gestational diabetes and gestational hypertension. These findings strengthen the validity of our conclusions and support the likelihood of an independent protective effect of supplementation. While our study provides valuable insights, it is not without limitations. The sample size, although adequate, could be expanded in future studies to enhance the generalizability of the findings. Additionally, we did not assess long-term maternal and child health outcomes, which could provide further evidence of the lasting benefits of calcium and vitamin D supplementation (27). Future research should explore these long-term effects and investigate the underlying biological mechanisms in more detail.

## Conclusion

This study demonstrates that combined calcium and vitamin D3 supplementation is associated with significantly improved maternal and neonatal health outcomes. The observed reductions in gestational diabetes, preeclampsia, gestational hypertension, and preterm birth, along with increases in neonatal birth weight and Apgar scores, highlight the importance of addressing nutritional deficiencies during pregnancy. Incorporating such interventions into routine prenatal care may enhance pregnancy outcomes and long-term child health. Further studies are warranted to explore long-term benefits and define optimal dosing strategies across diverse populations.

## Data Availability

The original contributions presented in the study are included in the article/Supplementary Material, further inquiries can be directed to the corresponding author.
